# γ-Glutamyltransferase enzyme activity of cancer cells modulates L-γ-glutamyl-p-nitroanilide (GPNA) cytotoxicity

**DOI:** 10.1038/s41598-018-37385-x

**Published:** 2019-01-29

**Authors:** Alessandro Corti, Silvia Dominici, Simona Piaggi, Eugenia Belcastro, Martina Chiu, Giuseppe Taurino, Simone Pacini, Ovidio Bussolati, Alfonso Pompella

**Affiliations:** 1 Department of Translational Research and New Technologies in Medicine and Surgery, University of Pisa, Via Roma 55, 56126 Pisa, Italy; 2 INSERM (French National Institute of Health and Medical Research), UMR 1260, Regenerative Nanomedicine (RMN), FMTS, 67000 Strasbourg, France; 3 Université de Strasbourg, Faculté de Pharmacie, 67000 Strasbourg, France; 4 Department of Medicine and Surgery, University of Parma, Via Volturno 39, 43125 Parma, Italy; 5 Department of Clinical and Experimental Medicine, University of Pisa, Via Roma 55, 56126 Pisa, Italy

## Abstract

L-γ-Glutamyl-p-nitroanilide (GPNA) is widely used to inhibit the glutamine (Gln) transporter ASCT2, but recent studies have demonstrated that it is also able to inhibit other sodium-dependent and independent amino acid transporters. Moreover, GPNA is a well known substrate of the enzyme γ-glutamyltransferase (GGT). Our aim was to evaluate the effect of GGT-mediated GPNA catabolism on cell viability and Gln transport. The GGT-catalyzed hydrolysis of GPNA produced cytotoxic effects in lung cancer A549 cells, resulting from the release of metabolite p-nitroaniline (PNA) rather than from the inhibition of Gln uptake. Interestingly, compounds like valproic acid, verapamil and reversan were able to increase the cytotoxicity of GPNA and PNA, suggesting a key role of intracellular detoxification mechanisms. Our data indicate that the mechanism of action of GPNA is more complex than believed, and further confirm the poor specificity of GPNA as an inhibitor of Gln transport. Different factors may modulate the final effects of GPNA, ranging from GGT and ASCT2 expression to intracellular defenses against xenobiotics. Thus, other strategies - such as a genetic suppression of ASCT2 or the identification of new specific inhibitors - should be preferred when inhibition of ASCT2 function is required.

## Introduction

Glutamine (Gln) is a nonessential aminoacid that plays a critical role in cell growth and proliferation. Several studies have focused on the high requirement of Gln by cancer cells and on its functions in supporting tumor growth^1,2^. Indeed, Gln is involved in purine, pyrimidine, non-essential amino acids (NEAAs) and lipid synthesis, and glutamine-derived carbon enters the tricarboxylic acid cycle (TCA). Moreover, Gln metabolism supports the synthesis of glutathione (GSH) and NADPH, which are both implicated in the maintenance of the intracellular redox state. Finally, the role played by Gln in tumor growth under hypoxic conditions and in autophagy-mediated prosurvival pathways was also demonstrated^1–5^. In this context, different studies have described an upregulation of high affinity glutamine transporters in cancer cells^4,6,7^, which is permissive for high rates of uptake and metabolism of the amino acid often observed in human cancers. Interestingly, a deregulated expression of MYC modulates both the metabolism of Gln and the expression of SLC1A5, the gene that encodes for the sodium-dependent Gln transporter Alanine-Serine-Cysteine Transporter 2 (ASCT2)^1,4^. A large number of human cancer cell lines are highly sensitive to Gln starvation, including those derived from pancreatic cancer, several subtypes of breast cancer, glioblastoma multiforme, acute myelogenous leukemia and non-small-cell lung cancer^4^. Therefore, it has been suggested that depriving cancer cells of Gln would be a feasible approach to limit tumor growth and to enhance the effects of some antitumor drugs^8^.

In this perspective, several studies focused on the ASCT2 transporter as a potential therapeutic target, and different approaches, including its inhibition, silencing or degradation upon pharmacologically-induced endoplasmic reticulum (ER) stress, were used^4,9,10^. Among the ASCT2 inhibitors, L-γ-glutamyl-p-nitroanilide (GPNA) is a widely used compound (*e.g*.^9,11–17^). However, recent studies have demonstrated that GPNA is also able to inhibit other Na^+^-dependent carriers, such as four members of the Na^+^-neutral amino acid transporters (SNAT) family (SNAT1, -2, -4, -5;^18^), and the Na^+^-independent leucine transporters LAT1/2^19^, thus raising the issue of the limited selectivity of GPNA in inhibiting ASCT2.

In addition to this, consideration is rarely given to the fact that GPNA is a well known chromogenic substrate of the enzyme γ-glutamyltransferase (GGT) and is commonly used to evaluate GGT-activity^20^. GGT catalyzes the hydrolysis of the gamma-glutamyl bond in GPNA, thus promoting the release of the chromogen *p*-nitroaniline (PNA). Interestingly, the mutagenicity and/or cytotoxicity of some gamma-glutamyl derivatives, including PNA, was demonstrated many years ago in *Salmonella typhymurium* strains^21,22^.

Taken together, we hypothesize that GGT contributes to the cytotoxic effects produced by GPNA.

## Materials and Methods

### Chemicals

Unless otherwise indicated, all reagents were from Sigma Chemical Co. (St. Louis, MO, USA).

### Cell lines and culture conditions

The human lung cancer cell line A549 (ICLC, Genova, Italy) were kindly provided by Dr. S. Cianchetti (University of Pisa, Pisa, Italy) and was routinely grown in RPMI 1640 medium supplemented with 2 mM L-glutamine and 10% foetal bovine serum (v/v). The BEAS-2B-derived clones were obtained by stable transfection with a vector containing the full-length cDNA of human GGT or the empty vector, as previously described^23^. BEAS-2B cells were routinely grown in DMEM medium supplemented with 2 mM L-glutamine, 10% foetal bovine serum (v/v) and 0.2 μg/mL G418 (Invitrogen). All cell lines were cultured at 37 °C in a 5%/95% CO_2_/air atmosphere.

### Cell treatments

Incubations were performed in RPMI 1640 supplemented with 2 mM L-glutamine, EGF (25 ng/ml) and 1% v/v of a growth factors cocktail (ITS; Corning, USA) including insulin, transferrin and selenium, as previously described^24^. The specific GGT inhibitor GGsToP^25^ was obtained from Tocris (UK); purified human GGT was obtained from Lee Biosolutions (St. Louis, MO, USA). The specific concentrations of each inhibitor were chosen among those commonly described in literature and are reported in the figure legends.

### Determination of GPNA conversion to PNA

Cells were plated at a density of 5,000 cells/well in 96-well plates and after 24 hrs treated with GPNA with/without the specific GGT inhibitor GGsToP in phenol-free RPMI 1640 supplemented as described above. GGT-mediated conversion of GPNA to PNA was spectrophotometrically monitored at 405 nm (Victor^3^ 1420 multilabel counter; Perkin-Elmer, Waltham, MA) over a 48-hrs incubation time.

### Glutamine uptake

For the measurement of Gln uptake, 10,000 cells/well were seeded in 96-well dishes and cultured for 24 hrs. The experiment was performed in Earle’s Balanced Salt Solution (EBSS, composition in mM: NaCl 117, NaHCO_3_ 26; KCl 5.3, CaCl_2_ 1.8, MgSO_4_·7H_2_O 0.81, choline phosphate 0.9, glucose 5.5, supplemented with 0.02% Phenol Red, kept at pH 7.4 under CO_2_ 5%). Cells were washed in EBSS and incubated in the same solution supplemented with L-[3,4-^3^H(N)]-Gln (2 mM, 15 μCi/ml (Amersham Biosciences)) for 1 min. Then, cells were washed with cold urea (300 mM) and the intracellular amino acid pool was extracted with absolute ethanol. The extracts were supplemented with 200 µl of scintillation fluid and counted with a scintillation spectrometer (Wallac Microbeta Trilux counter, Perkin-Elmer, Waltham, MA, USA). The non-saturable component of glutamine influx, estimated measuring glutamine uptake in the presence of 10 mM Gln, was subtracted to obtain saturable influx. Proteins were quantified with the Lowry method, and data were expressed as pmol/mg protein/min.

### Cell viability

Cytotoxicity was evaluated by the resazurin method following the manufacturer’s instructions (Sigma-Aldrich). Briefly, cells were plated at a density of 5,000 cells/well in 96-well plates and after 24 hrs treated as described in the figure legends. After further 48 hrs, 10 μl of resazurin dye solution were added to each well and, after an additional 2 hours of incubation, samples were analyzed fluorometrically (Victor^3^ 1420 multilabel counter; Perkin-Elmer, Waltham, MA). Cell viability was also evaluated with the Trypan blue exclusion test on cells plated at a density of 50,000 cells/well in 12-well plates and treated as described above.

### Apoptosis

Apoptosis was monitored with the Hoechst assay and with the Annexin V binding assay. For both assays, cells were plated in 96-well plates (5,000 cells/well) and, after 24 hrs, treated for 48 hrs as described in the figure legends.

For the Hoechst assay, the cells were stained with 5 μg/ml Hoechst 33342 (10 min, 37 °C), and both floating and adherent cells were analyzed by using a hemocytometer under a fluorescence microscope (Leica). Cells incorporating the Hoechst dye and showing typical morphological apoptotic features, such as chromatin condensation, were considered apoptotic cells, according to Schmid *et al*.^26^. Data were expressed as the apoptosis index ((apoptotic cells/total cells) × 100).

For the Annexin V assay, treated cells were stained using the annexin V–FITC fluorescence microscopy kit (BD Biosciences), according to the manufacturer’s instructions. Apoptotic cells were finally detected with a fluorescence microscope (Leica) equipped with an online image capture system (LeicaDFC320).

### Flow cytometry

For the analysis of DNA content and cell cycle by flow cytometry, cells were pelleted, washed twice with PBS, fixed with 70% v/v ethanol. At the time of flow cytometry analysis, cells were washed twice in PBS, incubated 1 h at 37 °C with 0.5 mg/ml RNAase A (Qiagen, Hilden, GER) and then stained for DNA content with propidium iodide (PI) (Miltenyi Biotec, Bergisch Gladbach, GER) at a final concentration of 10 μg/ml. A total of 10,000 events were collected using MACSQuant® Analyzer 10 (Miltenyi Biotec). Doublets discrimination was applied before DNA content analysis performed using the MACSQuantify® software (Miltenyi Biotec).

### ROS measurement

Intracellular reactive oxygen species (ROS) levels were assessed with 2′,7′ – dichlorofluorescin diacetate (DCFH-DA). After diffusion into cells, DCFH-DA is deacetylated by cellular esterases to a nonfluorescent compound, which is later oxidized by reactive compounds (*e.g*. ROS) to the highly fluorescent product 2′,7′ – dichlorofluorescein (DCF). Briefly, A549 cells were incubated with GPNA and PNA for 12 to 48 hrs. Cells were then loaded with DCFH-DA (4 µM final concentration) for 40 min at 37 °C in the dark and washed twice with PBS. After cell lysis, samples were centrifuged at 1000 *g* for 5 min at 4 °C and the supernatants were aliquoted in 96-well black plates. The fluorescence intensity was measured (filter settings: 485 nm excitation, 530 nm emission) using a multilabel counter (Wallac1420-Victor^3^, PerkinElmer, Waltham, MA).

### Western blot analyses

For Western Blot, samples were harvested in hypotonic lysis buffer (10 mM Tris–HCl, pH 7.8) additioned with protease inhibitors. For caspase-9 determinations both adherent and detached cells were collected. All samples were separated by 10% SDS-PAGE and incubated with a rabbit anti-GGT antibody directed against the C-terminal 20 amino acids of human GGT heavy chain prepared as described^27^, with a rabbit anti-ASCT2 (monoclonal 1:1000; Cell Signaling Technology, Danvers, MA, USA) or with a rabbit anti-caspase-9 antibody (1:200; Santa Cruz Biotechnology, Santa Cruz, CA, USA). A rabbit anti-actin antibody (1:1000; Cell Signaling) or a rabbit anti-glyceraldehyde 3-phosphate dehydrogenase (GAPDH, polyclonal 1:4000 Sigma) were also used as loading control. Visualization of protein bands was obtained using a horseradish peroxidase-conjugated anti-rabbit IgG antibody (Santa Cruz Biotechnology, Santa Cruz, CA, USA) and the ECL detection system (Roche, Basel, Switzerland). Bands were analyzed with a Bio-Rad ChemiDoc apparatus equipped with the QuantityOne software.

### Other determinations

GGT activity was determined according to Huseby and Stromme^28^. GSH determinations were performed using the enzymatic method described by Baker *et al*.^29^ adapted to the microtiter plate reader. Protein content was determined by using the Pierce BCA Protein Assay Kit (Thermo Fisher Scientific), following manufacturer’s instructions. A standard curve ranging from 0.025 to 1 mg/mL was built with bovine serum albumin to calculate protein concentration.

### Statistics

Statistical analysis of data was performed by Student’s t-test or one-way ANOVA with Newman–Keuls test for multiple comparisons, as detailed for each experiment.

## Results

### Cell sensitivity to GPNA

**Figure 1:**
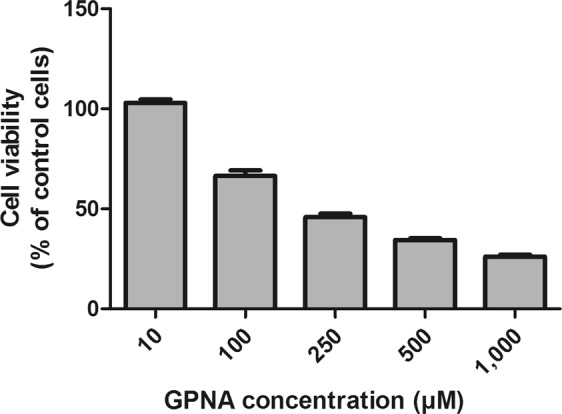
Effect of GPNA on cell viability. A549 cells were treated with increasing concentrations of GPNA for 48 hrs. Data are expressed as means ± s.d. of six values.

The human lung cancer cell line A549 is highly sensitive to Gln starvation. Indeed, when cells were incubated for 48 hrs in a Gln-free medium, a ~80% reduction of cell viability was detectable (data not shown). In a first set of experiments, we thus analyzed the effects of the glutamine analogue GPNA. As shown in Fig. 1, increasing concentrations of GPNA induced a dose-dependent decrease of cell viability, with an IC_50_ roughly corresponding to ~250 μM.

### Effect of GPNA catabolism on cell viability and apoptosis

**Figure 2:**
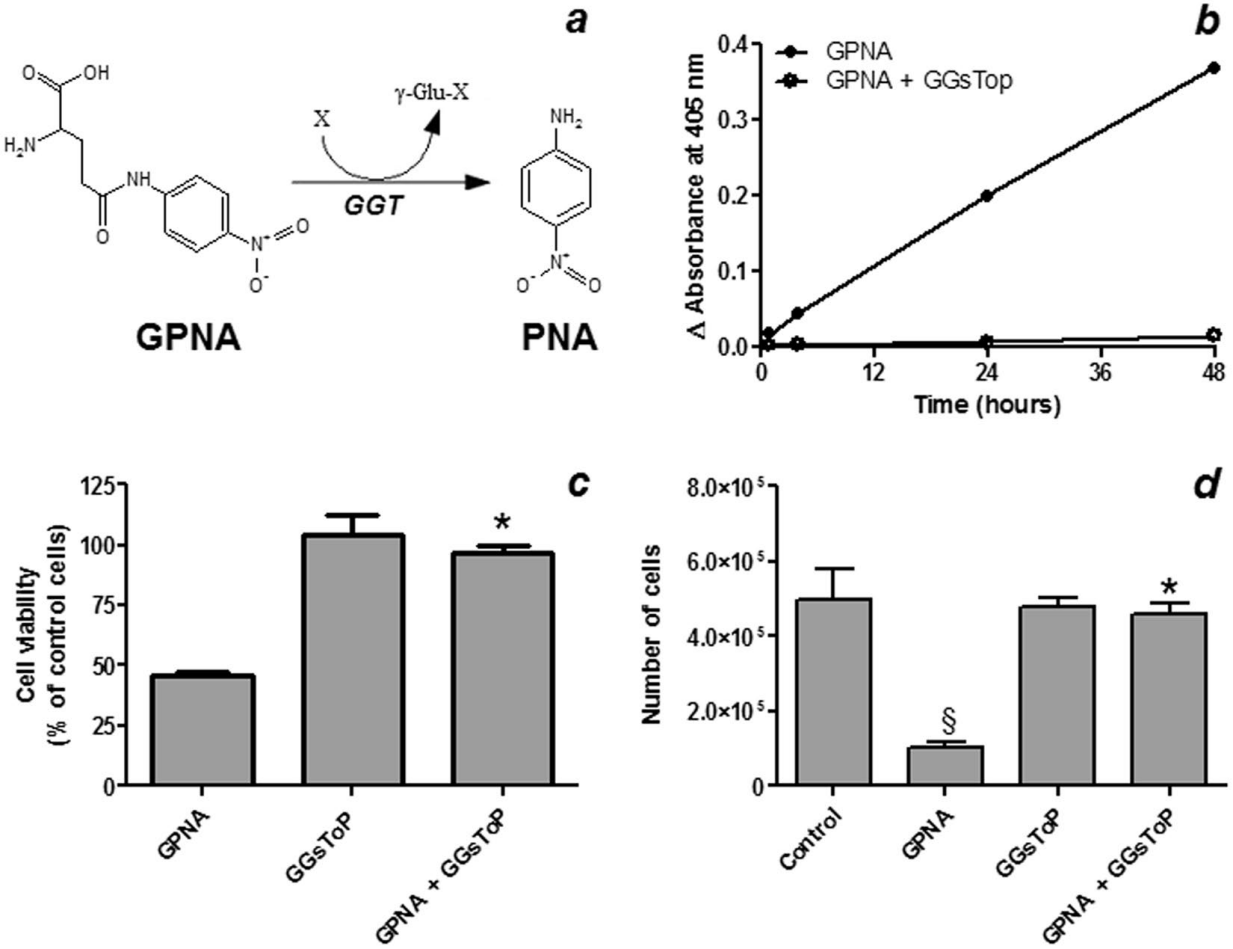
Effect of GPNA hydrolysis on cell viability. (**a**) GGT-mediated hydrolysis of GPNA to the chromogen PNA. (**b**) GPNA hydrolysis to PNA. A549 cells were incubated with 250 μM GPNA and the release of PNA was monitored at 405 nm for 48 hrs in the presence/absence of the specific GGT inhibitor GGsToP (20 μM). Data are expressed as means ± s.d. (**c**) Cell viability assessed with the resazurin method and (**d**) the Trypan blue exclusion test. A549 cells were incubated for 48 hrs with 250 μM GPNA. Where indicated GGsToP (20 μM) was added to the incubation mixture. Data are expressed as means ± s.d. of three to six values and were analyzed by one-way ANOVA with Newman–Keuls test for multiple comparisons. (*)p < 0.001 as compared to “GPNA”; (§)p < 0.01 as compared to “Control”.

**Figure 3:**
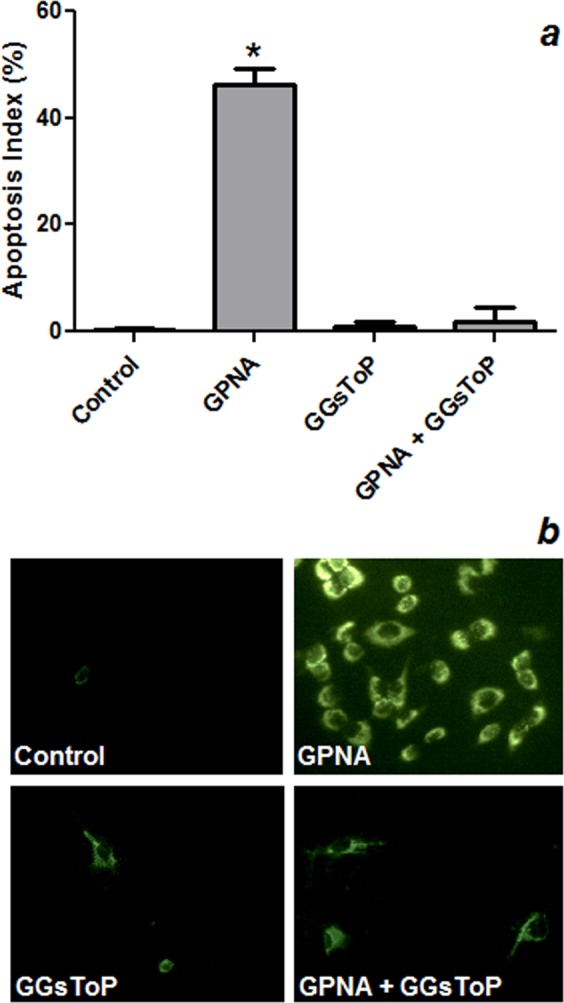
Effect of GPNA on apoptosis. A549 cells were incubated for 48 hrs with 250 μM GPNA or in a Gln-free medium. Where indicated 20 μM GGsToP was added to the incubation mixture. (**a**) Apoptosis index as determined by Hoechst staining. Data are expressed as means ± s.d. of six values and were analyzed by one-way ANOVA with Newman–Keuls test for multiple comparisons. (*)p < 0.0001 as compared to “Control”. (**b**) Immunofluorescence for Annexin V positivity of A549 cells treated with GPNA (250 μM), GGsToP (20 μM) or GPNA + GGsToP.

A549 cells express a GGT activity corresponding to ~30 mU/mg of protein. When cells were incubated with 250 μM GPNA, a time-dependent increase of absorbance at 405 nm, corresponding to the release of the chromogen PNA from GPNA, was observed (Fig. 2a,b). This effect was completely prevented by the specific GGT-inhibitor GGsToP (Fig. 2b). As judged by both resazurin method (Fig. 2c) and Trypan blue exclusion test (Fig. 2d) GGsToP was also able to completely prevent the cytotoxic effect of GPNA on A549 cells. Accordingly, when apoptosis was investigated by the Hoechst method (Fig. 3), a strong increase was detectable upon GPNA treatment for 48 hrs and, again, GGsToP completely prevented the effect observed. On the other hand, Gln starvation was able to induce only limited, not significant levels of apoptosis in A549 cells (data not shown).

### Effect of PNA on cell viability and apoptosis

**Figure 4:**
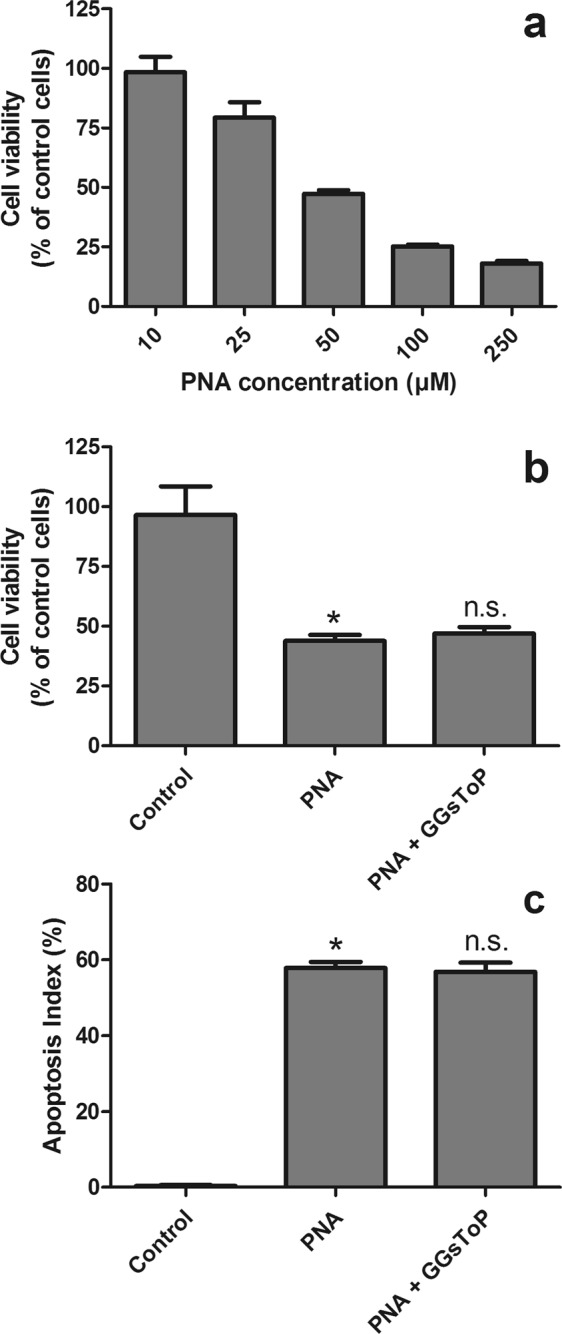
Effect of PNA on cell viability. (**a**) A549 cells were treated with increasing concentrations of PNA (10–250 μM) for 48 hrs. Data are expressed as means ± s.d. of six values. (**b**) A549 cells were incubated for 48 hrs with 50 μM PNA. Where indicated, GGsToP (20 μM) was added to the incubation medium. Viability (**b**) and apoptosis index (Hoechst staining, (**c**) were then evaluated. Data are expressed as means ± s.d. of three to six values (**b**) or six values (**c**) and were analyzed by one-way ANOVA with Newman–Keuls test for multiple comparisons. (*)p < 0.0001 as compared to “Control”; (n.s.) not significant as compared with “PNA”.

Based on the results described above, we then focused on the possible mechanisms involved in the cytotoxic effects of GPNA. The GGT-mediated hydrolysis of GPNA promotes the release of glutamic acid and PNA. As expected, glutamic acid, in the same range of concentrations used for GPNA (10–1000 μM), did not produce any appreciable effect on cell viability (see Supplementary Fig. S1). On the other hand, when A549 cells were incubated with increasing concentrations of PNA, a dose-dependent decrease of cell viability was detectable, with an IC_50_ corresponding to ~50 μM (Fig. 4a). The specific GGT-inhibitor GGsToP was not able to protect cells from PNA effects (Fig. 4b,c).

### Effect of GGT activity on GPNA sensitivity of BEAS-2B cells

**Figure 5:**
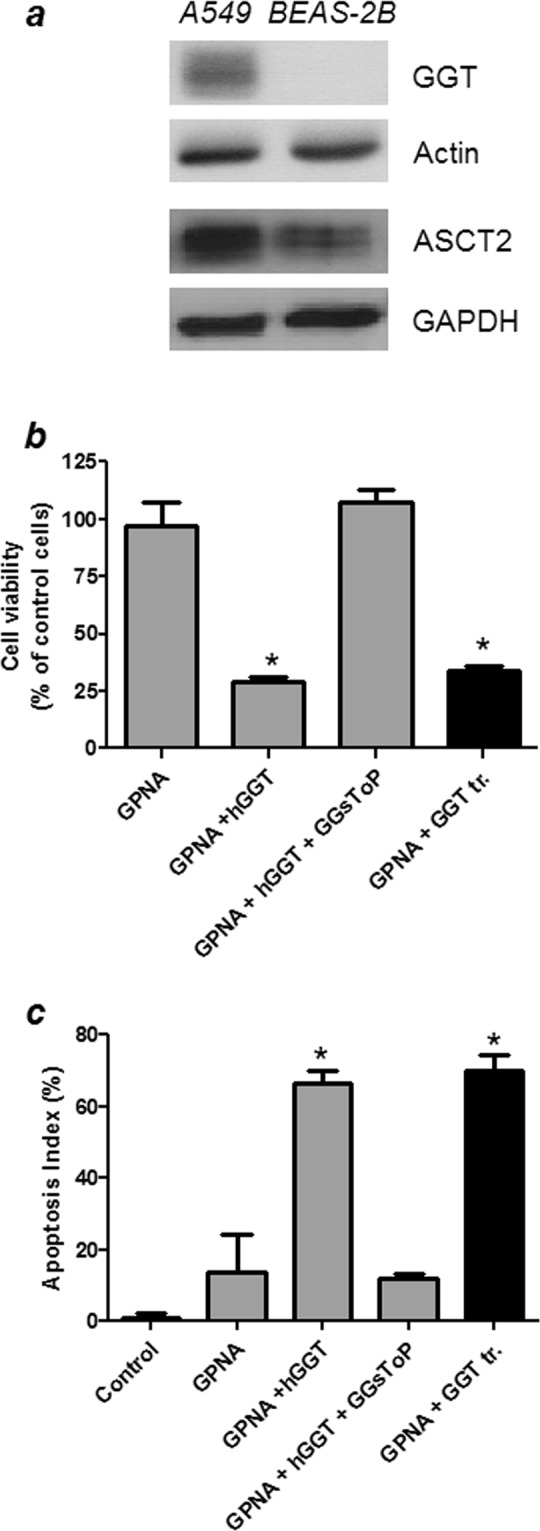
Effect of GPNA on BEAS-2B. (**a**) Western blot analysis of GGT and ASCT2 expression in A549 and BEAS-2B cells. Full-length blots are presented in Supplementary Fig. S6. (**b**,**c**) BEAS-2B cells were incubated for 48 hrs with 250 μM GPNA. Where indicated, 20 mU/ml of purified human GGT (hGGT) and 20 μM GGsToP were added to the incubation mixture. The “GGT tr.” sample corresponds to BEAS-2B cells stably transfected with human GGT (see 2.2 section in Material and Methods for further details). (**b**) Cell viability with the resazurin method; (**c**) Apoptosis index with Hoechst staining. Data are expressed as means ± s.d. of six values and were analyzed by one-way ANOVA with Newman–Keuls test for multiple comparisons. (*)p < 0.0001 as compared to “GPNA”.

In order to better understand the relationships between GGT and cell sensitivity to GPNA, the cell line BEAS-2B, endowed with a very low GGT activity (~0.3 mU/mg of protein), was used. As shown in Fig. 5a, western blot analysis confirmed that BEAS-2B cells had barely detectable GGT levels but, on the other hand, they also presented with a lower ASCT2 expression as compared to A549 cells. The sensitivity of BEAS-2B cells towards GPNA was very limited, as compared with A549 cells (see Supplementary Fig. S2), with an IC_50_ higher than 1 mM. Interestingly, when purified human GGT was added to the incubation medium or BEAS-2B cells were transfected with the full-length cDNA of human GGT, GPNA hydrolysis was increased (data not shown) and its cytotoxicity was strongly enhanced (Fig. 5b). This effect was associated with increased levels of apoptosis (Fig. 5c) and, again, it was prevented by the specific GGT-inhibitor GGsToP (Fig. 5 and data not shown). As observed for A549 cells, GGT addition/overexpression or the use of a GGT inhibitor did not modify BEAS-2B sensitivity to PNA (see Supplementary Fig. S3).

### GPNA and PNA effects on Gln uptake in A549 cells

**Figure 6:**
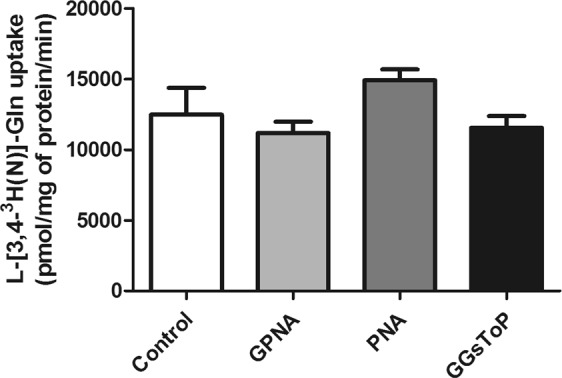
L-[3,4–^3^ H(N)]-Glutamine uptake by A549 cells. A549 were incubated with 2 mM radiolabeled Gln (15 μCi/mL; uptake time, 1 min), and cell-associated radioactivity determined as described under materials and methods (section 2.5). Where indicated, GPNA (250 μM), PNA (50 μM) or GGsToP (20 μM) were added to the uptake mixture. Data are expressed as means ± s.d. of five independent determinations.

The effect of GPNA and PNA on Gln uptake by A549 cells was then tested. Cells were incubated with 2 mM L-[3,4-^3 ^H(N)]-glutamine, *i.e*. the concentration of Gln commonly used in cell culture media, and in such conditions no significant effect of GPNA (250 μM), PNA (50 μM) or GGsToP (20 μM) on radiolabeled Gln uptake was detectable (Fig. 6).

### GPNA and PNA effects on cell cycle progression and apoptosis

**Figure 7:**
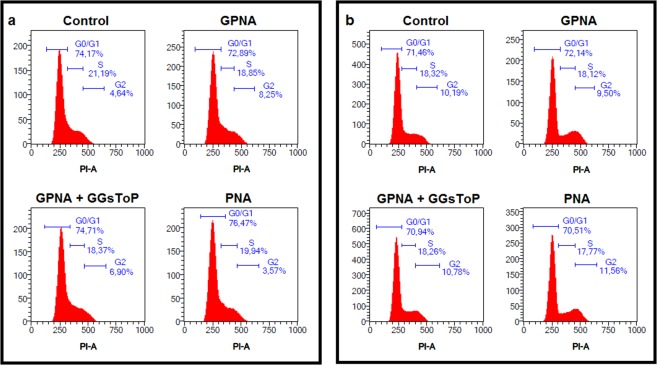
Effect of GPNA and PNA on cell cycle progression in A549. Cytofluorimetric analysis of cell cycle progression after (**a**) 24 hrs or (**b**) 48 hrs of incubation with GPNA (250 μM) or PNA (50 μM). Where indicated, GGsToP (20 μM) was added to the incubation medium. One representative experiment out of three is shown.

**Figure 8:**
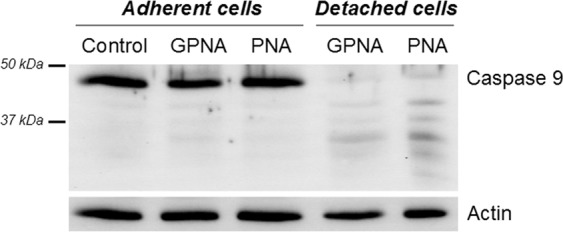
Caspase-9 processing in GPNA and PNA treated cells. Control A549 cells (lane 1) and cells treated with GPNA (250 μM) or PNA (50 μM) for 48 hrs were lysed and analysed by immunoblotting. Lanes 2 and 3: adherent cells; lanes 4 and 5: detached cells. Full-length blots are presented in Supplementary Fig. S7.

Another set of experiments was performed to study the mechanism of action of GPNA and PNA. As shown in Fig. 7, the cytofluorimetric analysis did not show any relevant effect on cell cycle progression after 24 or 48 hrs of incubation with GPNA or PNA. However, both compounds were able to induce apoptosis (see Figs 3 and 4). Consistently, as shown in Fig. 8, analysis of procaspase-9 in detached cells treated with GPNA or PNA, showed extensive processing with near complete disappearance of the p46 band and appearance of several different cleaved forms of caspase-9.

### Effects of GPNA and PNA on intracellular glutathione and ROS production

**Figure 9:**
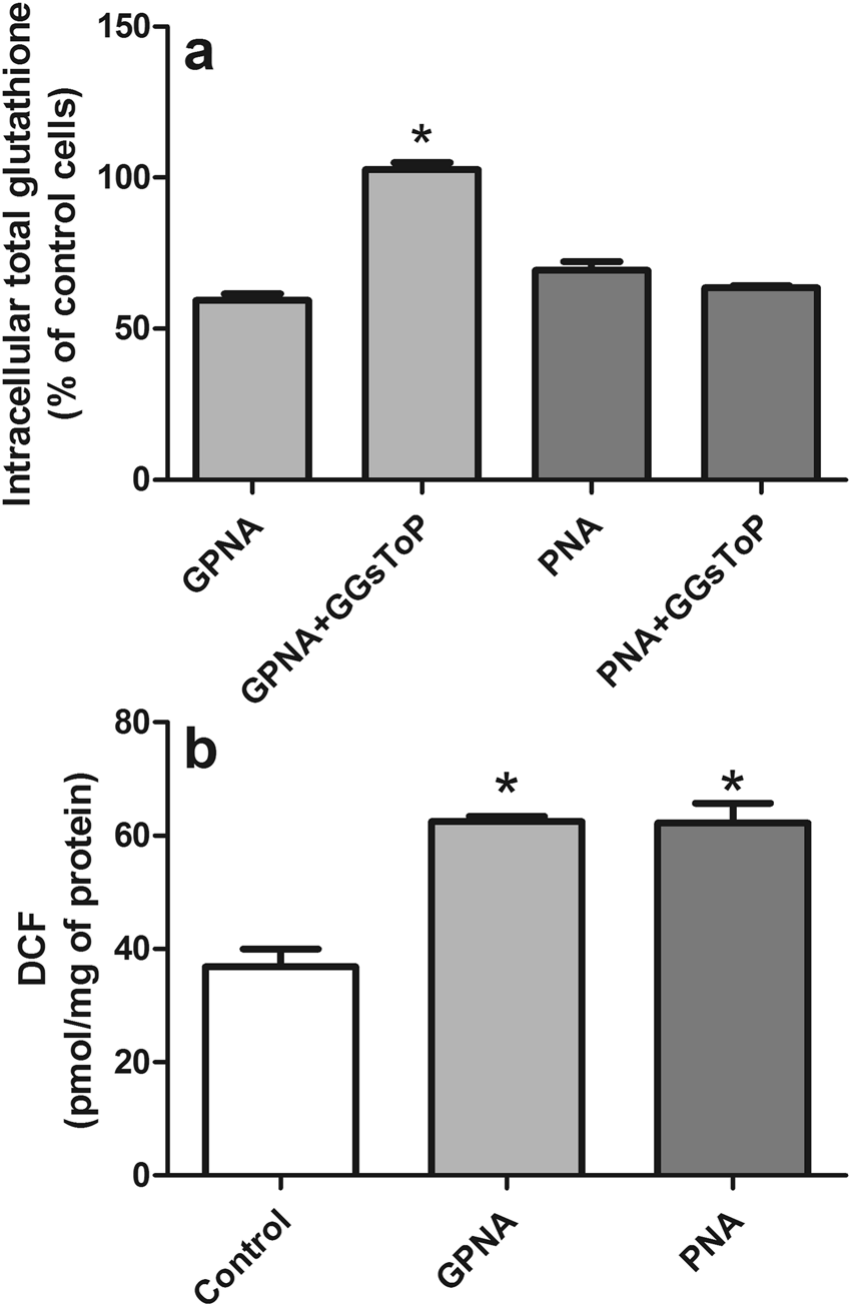
Effect of GPNA and PNA treatment on intracellular glutathione and ROS levels. A549 cells were treated with GPNA (250 μM) or PNA (50 μM) for 48 hrs. Where indicated, GGsToP (20 μM) was added to the incubation medium. At the end of the incubation total intracellular glutathione (**a**) and intracellular ROS (**b**) were measured. Data are expressed as means ± s.d. of three values and were analyzed by one-way ANOVA with Newman–Keuls test for multiple comparisons. For (**a**), (*)p < 0.01 as compared to “GPNA”. For (**b**), (*)p < 0.01 as compared to “Control”.

It was suggested that blocking Gln uptake could reduce the biosynthesis of glutathione, possibly resulting in increased intracellular reactive oxygen species (ROS;^24^). However, when both reduced (GSH) and oxidized (GSSG) intracellular glutathione were analyzed, we observed a significant reduction of total intracellular glutatione (GSH + GSSG) only at late times of incubation with GPNA and PNA (48 hrs; Fig. 9a), while no significant variation of GSSG was detectable (data not shown). Both treatments also produced increased levels of intracellular ROS, detected by the DCFH-DA method, but only at late incubation times (36–48 hrs; Fig. 9b). Indeed, shorter incubation times (12–24 hrs) did not produce any significant variation of both intracellular glutathione and ROS levels (see Supplementary Fig. S4).

### Effect of N-acetylcysteine on GPNA and PNA cytotoxicity

**Figure 10:**
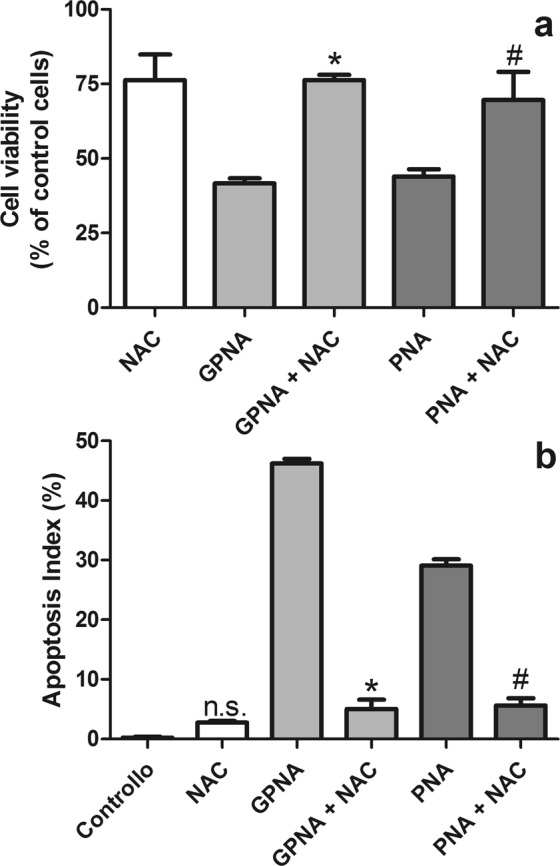
Effect of NAC on GPNA and PNA treatments. (**a**) Cell viability; (**b**) apoptosis. Cells were treated with GPNA (250 μM) or PNA (50 μM) for 48 hrs. Where indicated NAC (10 mM) was added to incubation media. Data are expressed as means ± s.d. of six values and were analyzed by Student’s t-test; (*)p < 0.0001 as compared to “GPNA”; (#)p < 0.0001 as compared to “PNA”.

N-acetylcysteine (NAC) supplementation was also proposed for reducing the toxic effects of the GPNA-mediated inhibition of Gln-uptake^12^. As shown in Fig. 10, 10 mM NAC induced a slight decrease (~25%) of cell proliferation after 48 hrs. On the other hand, the same treatment was also able to significantly reduce the cytotoxicity of both GPNA and PNA (Fig. 10a). This effect was associated with a significant reduction of apoptotic death (Fig. 10b).

### Mechanisms of PNA detoxification

**Figure 11:**
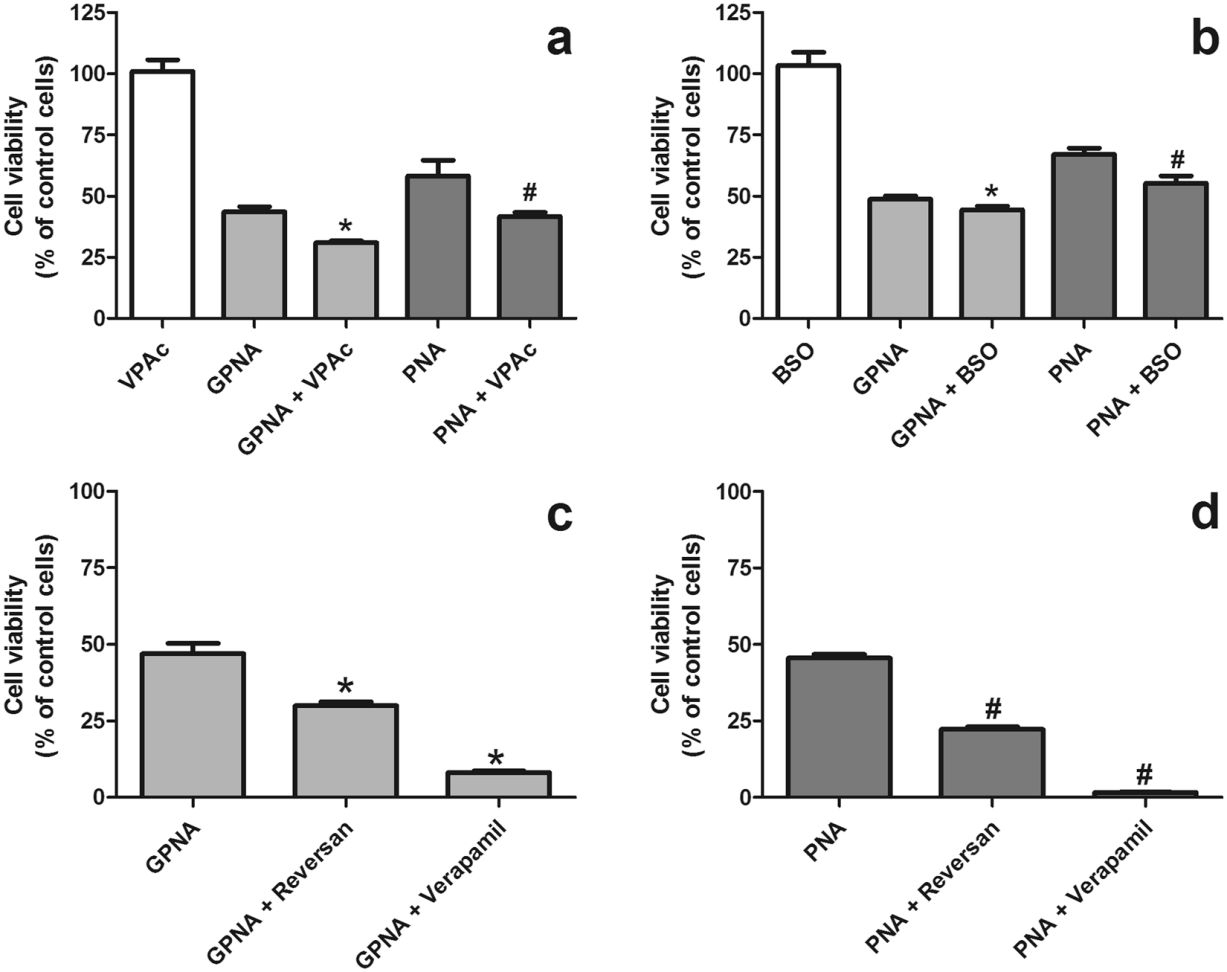
Effects of different inhibitors on GPNA and PNA-induced citotoxicity. Cells were treated with GPNA (250 μM) or PNA (50 μM) for 48 hrs. Where indicated valproic acid (VPAc; 250 μM), buthionine sulfoximine (BSO; 50 μM), reversan (10 μM) or verapamil (50 μM) were added to incubation media. Data are expressed as means ± s.d. of six values. Data were analyzed by Student’s t-test. (**a**) Effects of valproic acid, (*)p < 0.0001 as compared with “GPNA”; (#)p = 0.0001 as compared with “PNA”. (**b**) Effects of buthionine sulfoximine, (*)p < 0.001 as compared with “GPNA”; (#)p < 0.001 as compared with “PNA”. (**c**) Effects of reversan and verapamil on GPNA-induced citotoxicity, (*)p < 0.0001 as compared with “GPNA”. (**d**) Effects of reversan and verapamil on PNA-induced citotoxicity, (#)p < 0.0001 as compared with “PNA”.

In a last set of experiments we tried to elucidate the possible mechanisms involved in cellular sensitivity to PNA. As shown in Fig. 11a, valproic acid, a UDP-glucuronosyltransferase (UGTs) inhibitor, was able to significantly increase (p < 0.0001) both GPNA and PNA cytotoxicity. Also glutathione depletion by buthionine sulfoximine (BSO), a gamma-glutamylcysteine synthetase inhibitor, slightly increased the sensitivity of A549 to both compounds (Fig. 11b). However, the BSO concentration used here (50 μM) was able to induce a 40% decrease of intracellular glutathione only after 2 hrs and a stable > 90% decrease after 24 hrs and 48 hrs (see Supplementary Fig. S5). Finally, both reversan, an inhibitor of multidrug resistance-associated protein 1 (MRP1/ABCC1) and P-glycoprotein (ABCB1/MDR1; Pgp), and verapamil, a P-glycoprotein modulator, were able to significantly increase the cytotoxicity of both GPNA (Fig. 11c) and PNA (Fig. 11d).

## Discussion

Several types of cancer cells require large amounts of glutamine for rapid growth and are defined Gln-addicted. Different studies suggested that depriving Gln-addicted cells of Gln would be a feasible approach to limit tumor growth and to enhance the effects of some antitumor drugs^4,8^. In this perspective, the Na^+^-dependent transporter ASCT2 has been early identified as a potential therapeutic target and different strategies were proposed to inhibit its activity, thus producing an effective Gln deprivation.

To this purpose, GPNA has long been used as a “specific” ASCT2 inhibitor (*e.g*.^5,9,11–19^). However, a growing experimental evidence is unveiling its poor selectivity as a transport inhibitor^18,19^.

Besides these concerns about GPNA specificity, our study clearly demonstrates that, at least in part, GPNA effects may be the result of its hydrolysis by GGT. As expected^12^, GPNA was able to induce apoptosis in A549 cells (Figs 2 and 3) but this effect was completely inhibited by the specific GGT inhibitor GGsToP. Noteworthy, GGsToP was able to inhibit GPNA hydrolysis (and all the associated effects) without affecting Gln uptake (Fig. 6). A further confirmation of this interpretation came from experiments with the more GPNA-resistant BEAS-2B cell line (see also^12^), where an exogenously added GGT was also able to increase the cytoxicity of GPNA (Fig. 5). Interestingly, BEAS-2B cells are endowed with a sizable, although relatively low, expression of ASCT2 (Fig. 5).

GPNA is a well known GGT substrate which catalyzes the hydrolysis of its gamma-glutamyl bond and the consequent release of PNA^20^. Quite surprisingly, this feature is hardly taken into account when GPNA is used for ASCT2 inhibition. Depending on the GGT activity expressed by cells, GPNA concentration would decrease in the incubation medium thus producing a progressive loss of ASCT2 inhibition: this GGT-dependent, “limited” stability of GPNA can be easily detected in culture media where the chromogen PNA is released (Fig. 2b). Similarly, it was demonstrated that GPNA administration in rats results in high renal localization and release of PNA^30^ likely due to the high GGT activity expressed on the luminal surface of the proximal tubule cells of kidney^27^. However, this is only one part of the problem. Indeed, early studies demonstrated the mutagenicity and/or cytotoxicity of some gamma-glutamyl derivatives – including PNA – in *Salmonella typhymurium* strains^21,22^. According to these evidences, we observed that the cytotoxicity of GPNA on A549 can be mimicked by PNA treatments (Figs 4 and 8), although PNA does not affect Gln uptake (Fig. 6).

The GGT-dependent cytotoxicity of GPNA in A549 cells is further supported by the limited efficiency of GPNA as an inhibitor of Gln uptake. It was repeatedly demonstrated (*e.g*.^16,24^) and confirmed by us (data not shown) that when used at high concentrations (high micromolar/low millimolar range), GPNA inhibits the uptake of trace concentrations of Gln (low micromolar range). However, when the uptake assay is performed at the Gln concentration (2 mM) routinely used in cell culture media, cytotoxic concentrations of GPNA do not exert any significant inhibitory effect on Gln influx (Fig. 6). This result can be explained with the poor potency of GPNA as an inhibitor of ASCT2, whose external K_m_ for Gln was estimated to be as low as 40 μM^31^, thus raising the question of GPNA efficacy both *in vitro* and *in vivo*, where Gln concentrations were extimated to be ~0.7 mM in extracellular fluid and plasma^32^.

Our data indicate that the mechanism of action of PNA and GPNA in A549 cells are clearly overlapping, as also confirmed by their shared reduced effect on cell cycle progression (Fig. 7) and by the activation of the same caspase-9 dependent apoptotic pathway (Fig. 8). As already described for the sole GPNA^24^, both GPNA and PNA were able to increase intracellular ROS, even if at late incubation times (48 hrs; Fig. 9b), *i.e*. when apoptosis was clearly detectable (Figs 3 and 4). Although the limitations of ROS measurement by DCFH-DA are well known^33^, our data clearly indicate a similar behavior for GPNA and PNA and support the hypothesis that the effects produced are not the result of an inhibition of Gln uptake. It was observed that cytochrome c released from mitochondria during apoptosis is capable of oxidizing DCFH^34^ and thus the increase in DCF fluorescence that occurs during apoptosis of cells loaded with DCFH-DA has frequently been associated with enhanced oxidant production^33^. This could represent a possible explanation for the increased DCF fluorescence detectable upon GPNA and PNA treatments. Indeed, this interpretation seems to be supported by the lack of any GSSG increase along the 48 hrs incubations with both compounds (data not shown). Moreover, we observed a significant decrease of intracellular glutatione (GSH + GSSG) only at late incubation times (48 hrs; Fig. 9a), whereas it is known that GSH release is an active phenomenon regulating the redox signaling events involved in cell death activation and progression^35^. In this context, it was demonstrated that glutamate-L-cysteine ligase is a direct target of caspase 3^36^ and that apoptosis is associated with GSH depletion *via* the activation of a plasma membrane efflux transport (see^35^ for Refs.).

Other similarities between the effects produced by GPNA and PNA lie in the protective effect exerted by the antioxidant NAC on the effects of both compounds. NAC can modulate various metabolic pathways including those involved in apoptotic death^37^ and its ability in inhibiting the antigrowth effects of GPNA was assessed^12^. Indeed we observed that NAC, although at a quite high concentration (10 mM), was able to prevent the effects produced by both GPNA and PNA (Fig. 10).

In a last set of experiments we then evaluated which factors possibly modulate cell sensitivity to GPNA/PNA. Valproic acid is a compound known to inhibit the activity of enzymes such as cytochrome P450 and UDP-glucuronosyltransferases (UGTs), even if the largest inhibitory effect appears to be on drugs metabolized by the UGTs^38^. Interestingly, valproic acid (Fig. 11a) significantly increased the cytotoxic effects of both GPNA and PNA, thus suggeting the potential involvement of UGTs in the intracellular PNA detoxification. Intracellular glutathione could also play a role in the defense against PNA (Fig. 11b), but its effect seems minor. Indeed, a >90% decrease of intracellular glutathione with BSO was able to induce only a modest increase (5–10%) of both GPNA and PNA cytotoxicity.

Finally, also reversan and verapamil (Fig. 11c,d) induced a significant increase of GPNA/PNA cytotoxicity. Reversan is a multidrug resistance-associated protein 1 (MRP1/ABCC1) and P-glycoprotein (ABCB1/MDR1; Pgp) inhibitor^39^, while verapamil is a less specific Pgp modulator^40^. Our data suggest that the family of multidrug resistance (MDR) proteins could play a role in the resistance against GPNA/PNA and are in support of a mechanism of action alternative to the inhibition of Gln uptake.

All these data indicate that different cellular sensitivities to GPNA cannot be explained only with a different expression of ASCT2 or GGT. Indeed, while we did not observe any specific correlation between GGT expression and ASCT2 levels in a group of seven cell lines analyzed, including human liver cancer cells (HepG2, Huh6), multiple myeloma cells (OPM2, JJN3, RPMI 8226), lung cancer cells (A549) and immortalized human bronchial epithelial cells (BEAS-2B) (see Supplementary Fig. S6), we also demonstrated the lack of any direct or inverse correlation between GGT expression or ASCT2 levels and GPNA/PNA sensitivity (see Supplementary Table S1).

Hence the mechanism of action of GPNA is more complicated than expected, and several independent factors could contribute to the final outcome of treated cells. Indeed, (a) due to the lower ASCT2 affinity for GPNA, the relative Gln/GPNA concentrations could result in a limited or absent ASCT2 inhibition; (b) the limited specificity of GPNA for ASCT2 accounts for the inhibition of many other transporters for amino acids, with particular reference to essential amino acids^19^; (c) GPNA can be hydrolyzed (“activated”) by GGT and converted into the more cytotoxic PNA, so that both GGT expression and incubation times (*i.e*. kinetics of GPNA hydrolysis) can modulate the cytotoxic effects produced; (d) different cell types present with different levels of mechanisms implicated in the defense against xenobiotics, this possibly resulting in a higher/lower resistance against GGT-derived PNA. If the cancer cell exhibits a high sensitivity to PNA, then GPNA may effectively exert a significant, GGT-dependent toxicity.

The overlapping affinity of ASCT2 and GGT for GPNA is not an isolated case. The most potent GGT inhibitors are glutamine analogues and include acivicin, 6-diazo-5-oxo-L-norleucine and L-azaserine. However, they also inhibit glutamine metabolizing enzymes and their use was discontinued due to their high toxicity^4,41–43^. This feature should be taken into account when novel ASCT2 inhibitors are developed.

## Conclusions

In conclusion, our data, together with other recent findings from literature^18,19^, confirm that inhibitory effects of GPNA on cell viability are not easily attributable to the inhibition of Gln transport through ASCT2. Other strategies, such as a genetic suppression^9^, should be preferred when the role of ASCT2 transporter in a cellular model is studied. Moreover, these results should prompt investigations aimed to the identification of new specific ASCT2 inhibitors, an issue that, in spite of recent claims^44^, seems still unresolved^45^.

## Supplementary information

Supplementary figures, table and legends

## Data Availability

All data generated or analysed during this study are included in this published article and its Supplementary Information files.
