# Supplementary material for: γ-Glutamyltransferase enzyme activity of cancer cells modulates L-γ-glutamyl-p-nitroanilide (GPNA) cytotoxicity
Source: Sci Rep. 2019 Jan 29;9:891. doi: 10.1038/s41598-018-37385-x (PMC6351548; doi:10.1038/s41598-018-37385-x)
Supplement: Supplementary file 1 — Supplementary figures, table and legends [file 41598_2018_37385_MOESM1_ESM.pdf]

γ-Glutamyltransferase enzyme activity of cancer cells modulates L-γ-glutamyl-p-nitroanilide (GPNA) cytotoxicity.

Alessandro Corti (1), Silvia Dominici (1), Simona Piaggi (1), Eugenia Belcastro (1,2,3), Martina Chiu (4), Giuseppe Taurino (4), Simone Pacini (5), Ovidio Bussolati (4), Alfonso Pompella (1)

(1) Department of Translational Research and New Technologies in Medicine and Surgery, University of Pisa, Via Roma 55, 56126 Pisa, Italy; (2) INSERM (French National Institute of Health and Medical Research), UMR 1260, Regenerative Nanomedicine (RMN), FMTS, 67000 Strasbourg, France; (3) Université de Strasbourg, Faculté de Pharmacie, 67000 Strasbourg, France; (4) Department of Medicine and Surgery, University of Parma, Via Volturno 39, 43125 Parma, Italy; (5) Department of Clinical and Experimental Medicine, University of Pisa, Via Roma 55, 56126 Pisa, Italy.

<u>Legends to Supplementary Figures and Tables</u>

Figure S1 *Effect of glutamic acid on cell viability.* A549 cells were treated with increasing concentrations of glutamic acid for 48 hrs. Data are expressed as means ± s.d. of six values.

Figure S2 *Effect of GPNA on cell viability.* BEAS-2B cells were treated with increasing concentrations of GPNA for 48 hrs. Data are expressed as means ± s.d. of six values.

Figure S3 *Effect of PNA on BEAS-2B.* BEAS-2B cells were incubated for 48 hrs with PNA (150 μM) and where indicated GGsToP (20 μM) was added to the incubation mixture. (a) Cell viability and (b) apoptosis index as determined by Hoechst staining. Data are expressed as means ± s.d. of six values and were analyzed by one-way ANOVA with Newman–Keuls test for multiple comparisons.

Figure S4 *Effect of GPNA and PNA treatments on intracellular glutathione and ROS levels.* A549 cells were treated with GPNA (250 μM) or PNA (50 μM) for 12 or 24 hrs. At the end of the incubation, total intracellular glutathione (a) and intracellular ROS (b) were measured. Data are expressed as means ± s.d. of three values and were analyzed by one-way ANOVA with Newman–Keuls test for multiple comparisons.

Figure S5 *Glutathione depletion by buthionine sulfoximine (BSO).* A549 cells were treated with BSO (50 μM) for the indicated incubation times. Total intracellular glutathione was then measured as described in the Material and Methods section. Data are expressed as means ± s.d. of three values.

Figure S6 *GGT and ASCT2 expression in different cell lines.* Western blot analysis of GGT and ASCT2 expression in a group of seven cell lines including human liver cancer cells (HepG2, Huh6), multiple myeloma cells (OPM2, JJN3, RPMI 8226), lung cancer cells (A549) and immortalized human bronchial epithelial cells (BEAS-2B).

Figure S7 *Full-length blots of the cropped images shown in Figure 8.* Blots at lower exposure times are shown.

Table S1 *GGT activity, GPNA and PNA IC_{50} in different cell lines.* Data are expressed as means ± s.d. of three values.

Supplementary Figure S1

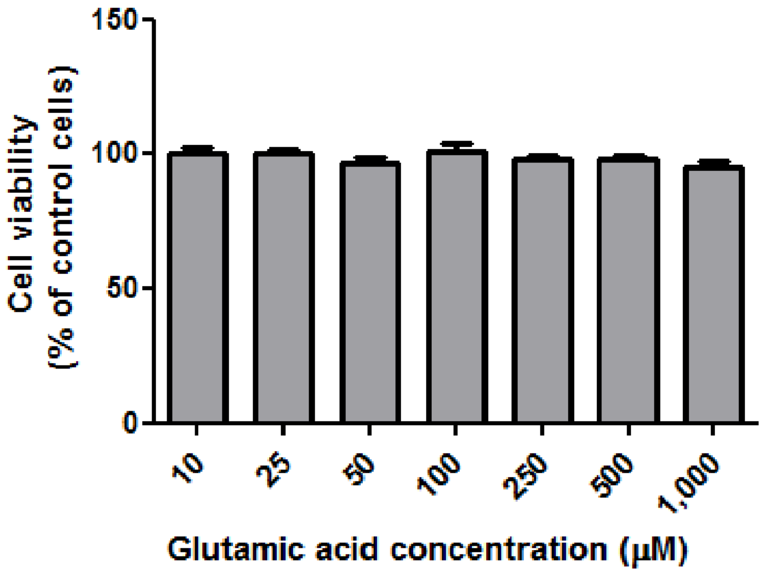

Supplementary Figure S2

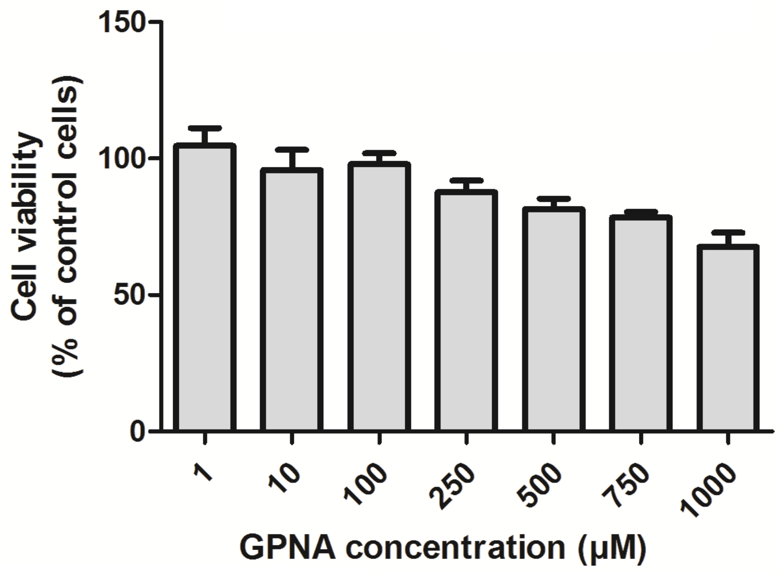

Supplementary Figure S3

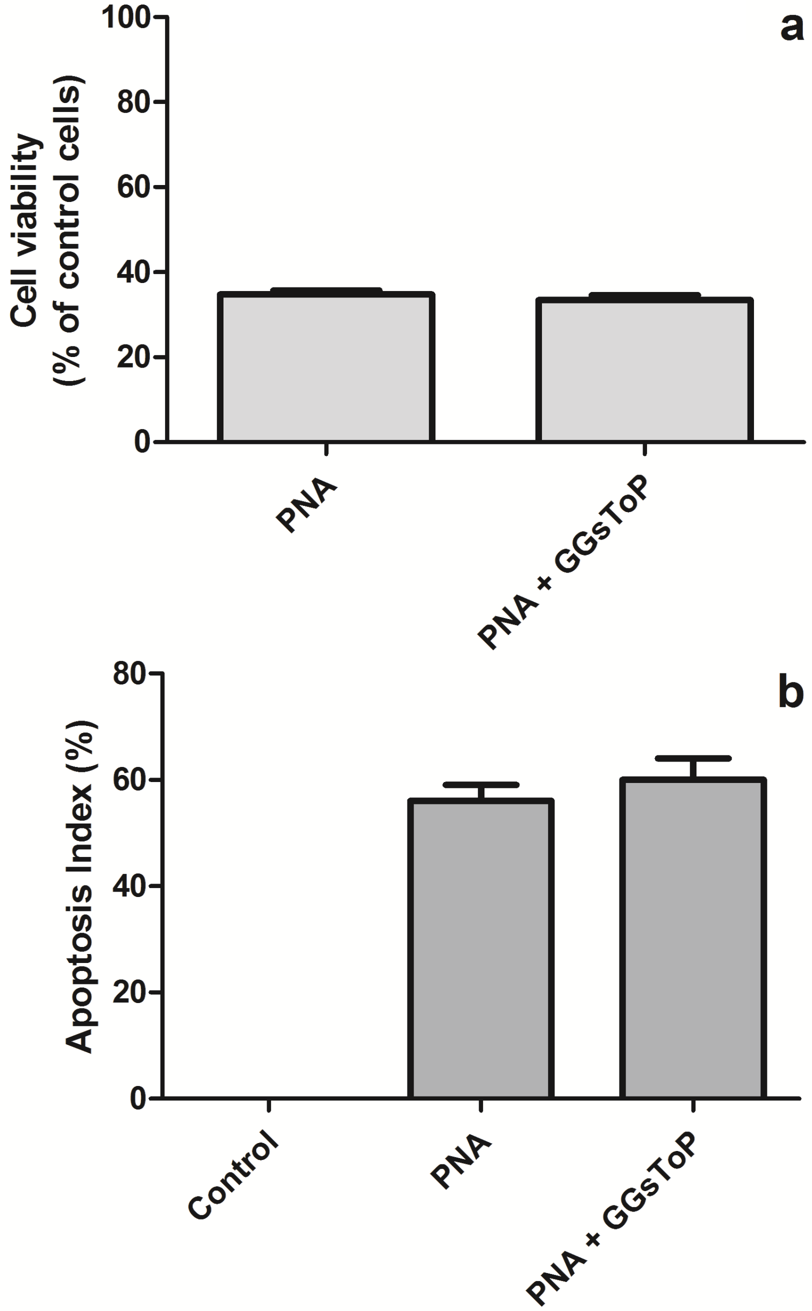

Supplementary Figure S4

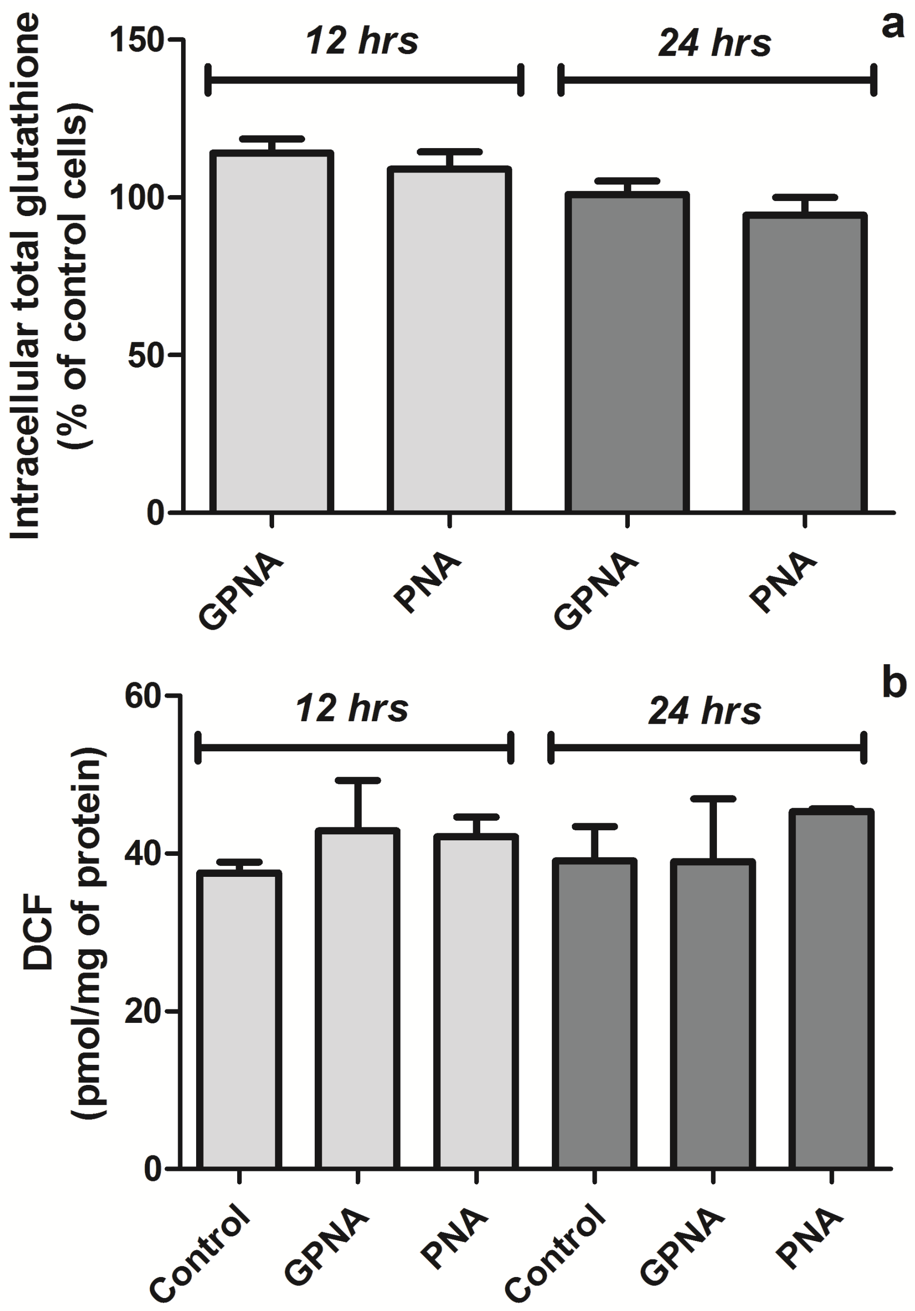

Supplementary Figure S5

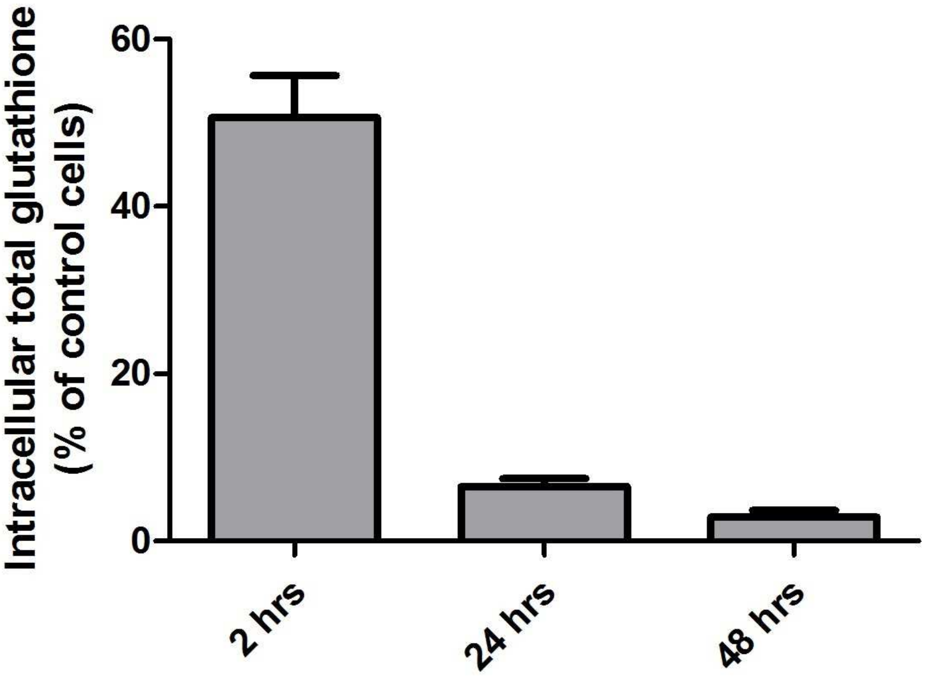

Supplementary Figure S6

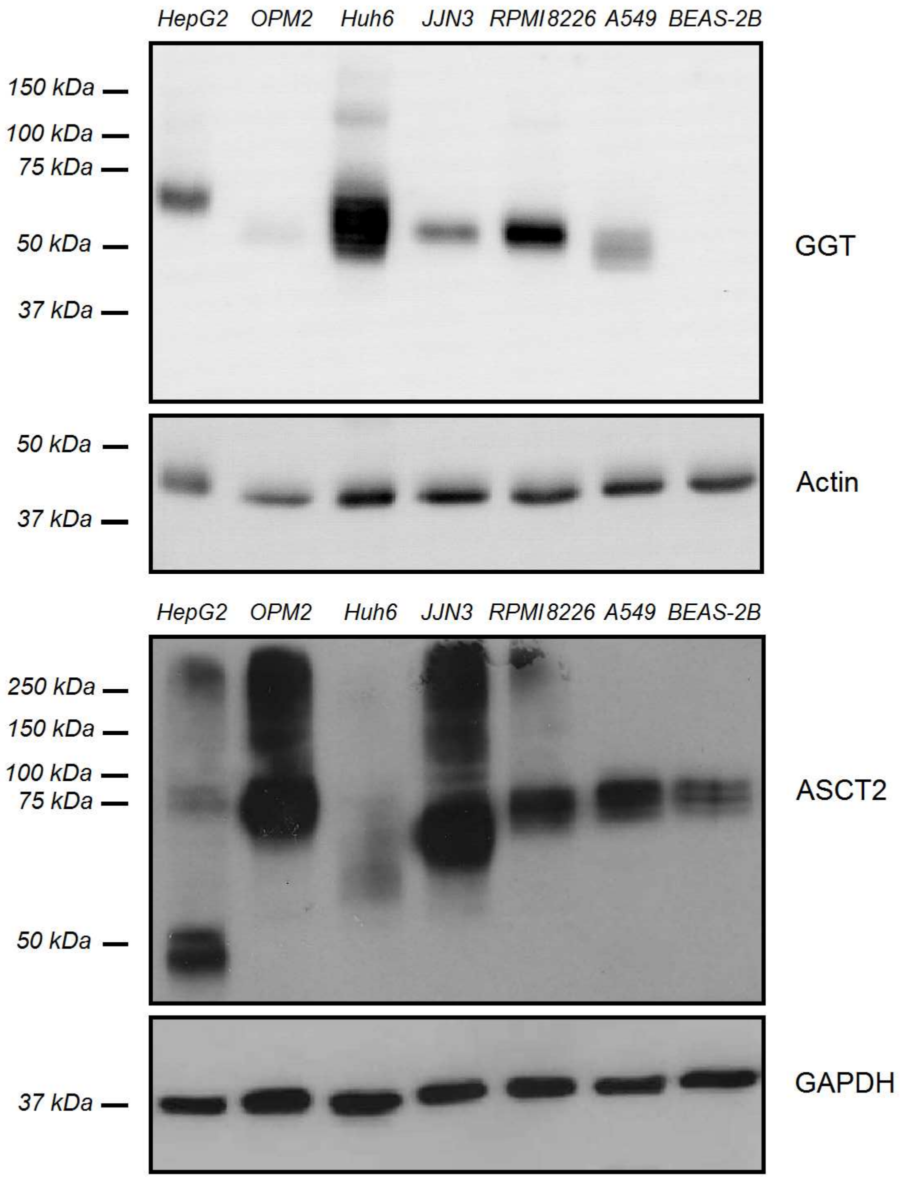

Supplementary Figure S7

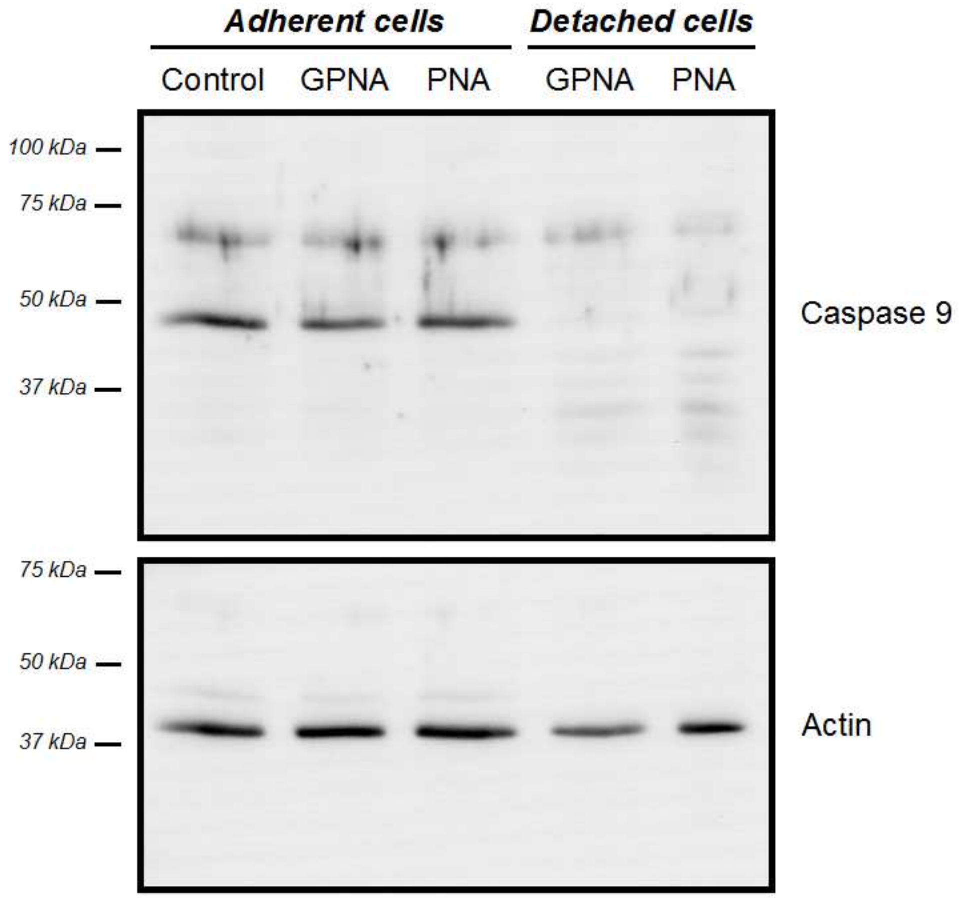

Supplementary Table S1

	HepG2	*OPM2*	*Huh6*	*JJN3*	*RPMI 8226*	*A549*	*BEAS-2B*
GGT activity (mU/mg of protein)	26.4 ± 1.1	13.9 ± 0.1	300.6 ± 6.6	36.3 ± 1.8	88.9 ± 0.9	32.6 ± 2.1	0.2 ± 0.1
GPNA IC_{50} (µM)	> 3000	~ 3000	>> 3000	~ 3000	~ 3000	250	> 1000
PNA IC_{50} (µM)	>> 600	>> 600	> 600	>> 600	>> 600	50	150
